# Clinical Characteristics of Rhinosinusitis in Children in a Tertiary Care Center

**DOI:** 10.7759/cureus.51236

**Published:** 2023-12-28

**Authors:** Abdullah M Alqahtani, Nawaf D Aljehani, Abdulazeez Alzailaie, Nawaf Alotaibi, Abdullah Alkhaldi, Jaber Alshammari

**Affiliations:** 1 College of Medicine, King Saud Bin Abdulaziz University for Health Sciences, Riyadh, SAU; 2 Division of Otolaryngology - Head & Neck Surgery, King Abdullah Specialized Children's Hospital, King Abdulaziz Medical City, National Guard Health Affairs, Riyadh, SAU

**Keywords:** sinusitis, rhinitis, pediatric rhinosinusitis, paranasal sinusitis, rhinosinusitis

## Abstract

Background: Rhinosinusitis (RS) is a term used in clinical practice to describe inflammation of the paranasal and nasal sinuses. This condition can be categorized based on the duration of symptoms into acute, subacute, and chronic RS. It is important to note that RS presents differently in pediatric patients compared to adults. In children, typical symptoms include cough, bad breath (halitosis), irritability, fatigue, and swelling around the eyes. This study aims to shed light on the prevalence and clinical characteristics of RS in the pediatric age group.

Methods: This retrospective cohort study was conducted at King Abdullah Specialized Children’s Hospital (KASCH) in Riyadh, Saudi Arabia, which is a tertiary care center under the authority of the Ministry of National Guard Health Affairs (MNGHA) in Saudi Arabia, using the medical records of all patients diagnosed with RS between 2019 and 2022.

Results: In this study, 345 pediatric patients with RS were examined. A significant portion (n = 106, 30.7%) were older than 12 years, and males made up the majority (n = 210, 60.9%). Chronic RS without nasal polyps prevailed (n = 299, 86.7%), mainly affecting the maxillary sinus (n = 200, 58%). Notably, 29% (n = 100) were diagnosed after age 12. Key symptoms included nasal congestion (n = 233, 67.5%), nasal discharge (n = 202, 58.6%), and facial discomfort (n = 191, 55.4%). Most (n = 314, 91%) received medical treatment, resulting in improvement for 78.8% (n = 272). Of those not improving (n = 73, 21.2%), 47.9% (n = 35) received medical management, and the rest underwent surgery, primarily functional endoscopic sinus surgery (n = 38, 52.1%).

Conclusion: RS is a common condition affecting children, with symptoms like nasal obstruction, discharge, and facial discomfort. Chronic RS, particularly in the maxillary sinus, is the most prevalent type. Medical treatment was the first choice and generally effective, but when needed, surgical intervention, mainly functional endoscopic sinus surgery, was pursued.

## Introduction

Rhinosinusitis (RS) can be clinically defined as an inflammatory process of the paranasal and nasal sinuses [1]. Rhinitis and sinusitis often occur together in most people. As a result, the preferred term to be used to describe this condition is "rhinosinusitis" [2]. The classification of RS based on the duration of clinical symptoms is as follows: acute RS, which lasts up to one month; subacute RS, which lasts between one and three months; and chronic RS, which lasts longer than three months [3]. According to the European Position Paper on Rhinosinusitis and Nasal Polyps, RS in children is diagnosed by the presence of two or more out of four characteristic symptoms, including nasal blockage, nasal discharge, facial pain, and cough [4].

RS in the pediatric age group differs significantly from that in adults. Children commonly exhibit a different set of symptoms, which include cough, halitosis, irritability, fatigue, and eye swelling. In addition, they may experience a thick nasal or post-nasal discharge that is yellow-green in color [5-7]. This may differ in adults, as they usually present with hyposmia instead of cough [4]. There are several risk factors for RS, including repeated upper respiratory tract infections (URTIs), nasal allergies, exposure to tobacco, and ventilation disorders of the sinuses, such as hypertrophy of nasal turbinate, nasal polyps, and deviation of nasal septum [8]. RS is considered a major burden on society with 30 million people in the United States being diagnosed with sinusitis annually, which costs the healthcare system about six billion dollars. Although the exact incidence of sinusitis in children is unknown, most children have six to eight episodes of URTIs in their first 10 years of life, and about 5-10% of those are complicated with sinusitis [9,10].

Although RS represents a rising issue globally, it is difficult to accurately estimate its prevalence. According to a study done in the USA that followed children from birth till the age of eight years, the prevalence of sinusitis was 13.1% with most cases being associated with allergic rhinitis [11,12]. Another study estimated the prevalence of RS in the pediatric age group to be 15-20% [10]. In Saudi Arabia, studies regarding the prevalence and clinical presentation of RS in pediatric patients are scarce. A study done in Aseer reported that 30 patients out of the 100 patients included had a previous history of RS with 38% having a positive family history [13]. It is worth noting that this is the only study that has been conducted thus far, addressing the issue of sinusitis in Saudi Arabia. However, it is important to acknowledge that there have been a few studies conducted on sinusitis in the pediatric age group, albeit with a narrower focus on specific types of sinusitis, such as allergic fungal sinusitis. These studies have provided valuable insights into certain aspects of pediatric sinusitis but do not comprehensively cover the broader spectrum of RS.

Due to the lack of local studies on sinusitis in the pediatric age group, especially in the central region, this study aims to shed light on the prevalence and clinical characteristics of RS in the pediatric age group in King Abdullah Specialized Children’s Hospital, Saudi Arabia. This study also investigates the relationship between treatment and outcome in accordance with the site of sinusitis. Moreover, given that our hospital is one of the largest and most prominent healthcare facilities in the country, the data collected through a comprehensive review of electronic records via the medical record system (BESTCare) can offer a comprehensive and accurate description of the prevalence, presentation, and outcomes of RS in Saudi Arabia contributing to the existing body of knowledge.

## Materials and methods

Following the Institutional Review Board (IRB) approval (NRC22R/649/12) by King Abdullah International Medical Research Center (KAIMRC), we conducted a retrospective cohort study using the medical records of all patients diagnosed with RS at King Abdullah Specialized Children’s Hospital (KASCH) in Riyadh, Saudi Arabia, which is a tertiary care center under the authority of the Ministry of National Guard Health Affairs (MNGHA) in Saudi Arabia. The inclusion criterion was all pediatric patients (aged ≤ 14 years) with a diagnosis of RS according to the European Position Paper on Rhinosinusitis and Nasal Polyps 2020 from January 2019 to December 2022. All patients who did not follow up were excluded. All patients who met the inclusion criterion were selected in this study. The sampling technique that was used in this research was the non-probability consecutive sampling technique.

Our aim is to evaluate the prevalence and clinical characteristics of children with RS by documenting demographic data, clinical characteristics, treatment, and outcomes. Furthermore, documentation of patients’ type and site of sinusitis was taken into consideration, which was correlated in data analysis in accordance with treatment and outcomes. Data from the patients’ electronic records via the medical record system (BESTCare), were entered into an Excel sheet (Microsoft Corporation, Redmond, WA) by the investigators/authors and verified by a person independent from the data entry person. Complete confidentiality was maintained during the collection and analysis of the data. Moreover, to ensure privacy and avoid harming patients, patients’ medical data were kept anonymous. No medical record numbers or names were taken during data collection. After all data corrections and cleaning, the data underwent statistical analysis. Microsoft Excel was used for data entry and management. Data analysis was carried out using Statistical Package for Social Sciences (SPSS; IBM Corp., Armonk, NY).

Descriptive and analytical statistics were applied to the data. Categorical data, such as age group, gender, patient type, type of sinusitis, and site, were presented as frequencies and percentages. Numerical data, such as age, BMI, and age group at diagnosis, were presented as mean and standard deviation (SD). A chi-square test was performed on categorical data. Student's t-test was performed for numerical data. For all analyses, p-value < 0.05 was considered statistically significant in this study.

## Results

This study reviewed 345 pediatric patients with the diagnosis of RS. As seen in Table 1, 30.7% were aged more than 12 years old, with males being dominant (60.9%). Most of the patients (80.3%) had normal BMI levels. A total of 60.9% were outpatients. The most common type of sinusitis was chronic RS without nasal polyps (86.7%), with the maxillary sinus being the most common site involved (58%). A total of 29% who had been diagnosed with sinusitis were older than 12 years.

**Table 1:** Demographic data and clinical characteristics

Study variable	N (%)
Age group	
1-3 years	11 (03.2%)
4-6 years	46 (13.3%)
7-9 years	91 (26.4%)
10-12 years	91 (26.4%)
>12 years	106 (30.7%)
Gender	
Male	210 (60.9%)
Female	135 (39.1%)
BMI level	
Underweight (<5^th ^percentile)	16 (04.6%)
Normal (5^th^-85^th ^percentile)	277 (80.3%)
Overweight (85^th ^-<95^th^ percentile)	34 (09.9%)
Obese (>95^th^ percentile)	18 (05.2%)
Patient type	
Outpatient (OP)	210 (60.9%)
Inpatient (IP)	17 (04.9%)
Emergency (ED)	118 (34.2%)
Type of sinusitis	
Acute rhinosinusitis	30 (08.7%)
Chronic rhinosinusitis without nasal polyps	299 (86.7%)
Chronic rhinosinusitis with nasal polyps	16 (04.6%)
Site	
Maxillary	200 (58.0%)
Ethmoidal	64 (18.6%)
Frontal	12 (03.5%)
Sphenoid	11 (03.2%)
Pansinusitis	58 (16.8%)
Age group at diagnosis	
1-3 years	13 (03.8%)
4-6 years	49 (14.2%)
7-9 years	91 (26.4%)
10-12 years	92 (26.7%)
>12 years	100 (29.0%)

In Table 1, the most common clinical presentation of RS was nasal obstruction (67.5%), followed by discharge from the nose (58.6%) and facial pain/pressure/discomfort (55.4%).

In Table 2, nearly all patients (91%) received medical management, with 50.4% receiving antibiotics. Patients who used intranasal steroids constituted 56.2% and only 5.8% underwent surgical intervention. Other patients received medication, with normal saline (12.8%) and loratadine (10.1%) being the most common. A total of 42.9% had a duration of follow-up of more than five months. The prevalence of patients who showed improvement after the treatment was 78.8%. The rest of the patients did not show improvement (21.2%). Among those who did not improve (n = 73), 52.1% needed surgical management, with functional endoscopic sinus surgery (FESS) being the procedure of choice.

**Table 2:** Treatment and outcomes

Treatment and outcomes	
Study variable	N (%)
Medical management	
Yes	314 (91.0%)
No	29 (08.4%)
Not done	2 (0.60%)
Antibiotics	
Yes	174 (50.4%)
No	167 (48.4%)
Not done	4 (01.2%)
Intranasal steroids	
Yes	194 (56.2%)
No	147 (42.6%)
Not done	4 (01.2%)
Surgical intervention	
Yes	20 (05.8%)
No	318 (92.2%)
Not done	7 (02.0%)
Type of treatment	
Acetaminophen	13 (03.8%)
Ibuprofen	22 (06.4%)
Loratadine	35 (10.1%)
Functional endoscopic sinus surgery (FESS)	20 (05.8%)
Normal saline	44 (12.8%)
Salbutamol	4 (01.2%)
Xylometazoline	21 (06.1%)
Others	5 (01.4%)
None	181 (52.5%)
Duration of follow-up	
≤5 months	197 (57.1%)
>5 months	148 (42.9%)
Improvement	
Yes	272 (78.8%)
No	73 (21.2%)
Outcome if not improved	
Surgical management	38 (52.1%)
Medical management	35 (47.9%)
Specific management	
Functional endoscopic sinus surgery (FESS)	38 (52.1%)
Intranasal steroids	17 (23.3%)
Antibiotics	4 (05.5%)
Nasal endoscopy	1 (01.4%)
Loratadine	2 (02.7%)
Others	11 (15.1%)

When measuring the relationship between the patient type among the treatment and outcome (Table 3), it was observed that the prevalence of patients who received antibiotics (p < 0.001) and underwent surgical intervention (p = 0.003) was significantly more common among inpatients while the prevalence of patient who received intranasal steroid was significantly more common among outpatients (p < 0.001).

**Table 3:** Relationship between patient type according to treatment and outcomes

Factor	Status	Outpatient, N (%)	Inpatient, N (%)	Emergency, N (%)	p-value
Medical management	Yes	190 (90.9%)	15 (88.2%)	109 (93.2%)	0.629
	No	19 (09.1%)	2 (111.8%)	8 (06.8%)	
Antibiotics	Yes	75 (36.2%)	13 (76.5%)	86 (73.5%)	<0.001
	No	132 (63.8%)	4 (23.5%)	31 (26.5%)	
Intranasal steroids	Yes	144 (69.9%)	8 (47.1%)	42 (35.9%)	<0.001
	No	63 (30.4%)	9 (52.9%)	75 (64.1%)	
Surgical intervention	Yes	14 (06.8%)	4 (25%)	2 (01.7%)	0.003
	No	192 (93.2%)	12 (75%)	114 (98.3%)	

In Table 4, it was revealed that the prevalence of patients who underwent surgical intervention was significantly more common among the non-maxillary group (p = 0.011).

**Table 4:** Relationship between site of sinusitis according to treatment and outcomes

Factor	Status	Maxillary, N (%)	Non-maxillary, N (%)	p-value
Medical management	Yes	180 (90.9%)	134 (92.4%)	0.621
	No	18 (09.1%)	11 (7.6%)	
Surgical intervention	Yes	6 (03.1%)	14 (09.7%)	0.011
	No	188 (96.9%)	130 (90.3%)	

In Table 5, significant improvements were seen in younger patients (p = 0.001), emergency patients (p = 0.001), and patients with acute RS (p = 0.001).

**Table 5:** Relationship between improvement according to demographic and type of sinusitis

Factor	Status	Improvement, N (%)		No improvement, N (%)	p-value
Age group	<10 years	129 (47.4%)	19 (26%)		0.001
	>10 years	143 (52.6%)	54 (74%)		
Gender	Male	164 (60.3%)	46 (63.0%)		0.688
	Female	108 (39.7%)	27 (37.0%)		
Patient type	Outpatient	153 (56.3%)	57 (78.1%)		0.001
	Inpatient	13 (04.8%)	4 (05.5%)		
	Emergency	106 (39.0%)	12 (16.4%)		
Sinusitis type	Acute rhinosinusitis	29 (10.7%)	1 (01.4%)		0.001
	Chronic rhinosinusitis without nasal polyp	235 (86.4%)	64 (87.7%)		
	Chronic rhinosinusitis with nasal polyp	8 (02.9%)	8 (11.0%)		

In Table 6, it was found that there was no significant relationship observed between RS type in terms of mucous culture, medical management, antibiotics, steroids, and surgical intervention (all p > 0.05).

**Table 6:** Relationship between type of sinusitis according to treatment and outcomes RS: rhinosinusitis.

Factor	Status	Acute RS, N (%) (n = 30)	Chronic RS, N (%) (n = 315)	p-value
Mucous culture	Positive	0 (0%)	7 (35%)	0.119
	Negative	5 (100%)	13 (65%)	
Medical management	Yes	26 (89.7%)	288 (91.7%)	0.702
	No	3 (10.3%)	26 (8.3%)	
Antibiotics	Yes	18 (62.1%)	156 (50%)	0.214
	No	11 (37.9%)	156 (50%)	
Steroids	Yes	13 (44.8%)	181 (58%)	0.170
	No	16 (55.2%)	131 (42%)	
Surgical intervention	Yes	2 (6.9%)	18 (5.8%)	0.815
	No	27 (93.1%)	291 (94.2%)	

## Discussion

The magnitude of RS as a growing problem in society makes it challenging to accurately assess its prevalence. In the literature, the number of studies describing the prevalence of RS in pediatric patients, especially in Saudi Arabia, is scarce. Thus, our study, which included 345 patients, aims to shed light on the prevalence and clinical presentation of RS to reach early diagnosis and timely appropriate medical and surgical intervention.

In the pediatric age group, patients with RS can vary in their clinical presentation. The presence of characteristic symptoms such as nasal blockage, nasal discharge, facial pain, and cough raises the diagnostic probability of RS. In our study, we found that the most common clinical presentation of RS in children was nasal obstruction, followed by nasal discharge and facial pain/discomfort, which is consistent with the European Position Paper on Rhinosinusitis and Nasal Polyps published in 2020 [14]. In contrast, other studies have found that rhinorrhea (96%) and cough (88%) were the most common presenting symptoms in RS [15]. Moreover, in a study that was done in Aseer, they found that fever followed by red eye, ringing nose, cough, and headache with fatigue were the most common symptoms, which was not consistent with our study as fever was only encountered in 17% of the cases [13].

Out of the 345 pediatric patients, 1/3 of patients were aged more than 12 years old, with males being more (60.9%). RS can vary in its types; our study reported chronic RS without nasal polyps to be the most common type followed by acute RS. Another study demonstrated that chronic RS was the most common cause; however, the following type was allergic rhinitis [16]. The most affected sinuses in our study were the maxillary sinus (more than half), followed by the ethmoidal sinus. Similarly, a study conducted in Poland found that the most common site was the maxillary. Moreover, ethmoid and sphenoid sinuses were more likely to be involved in children with inhalant allergies [17]. The most isolated pathogens out of the seven patients in our study were fungi, *Staphylococcus aureus*, and *Haemophilus influenzae*. Other studies frequently mention rhinovirus, parainfluenza, *Streptococcus pneumoniae*, and *Haemophilus influenzae*, with *Staphylococcus aureus* being the most common among them [18].

In terms of treatment, nearly all patients received medical management. This is consistent with the literature as medical therapy is the mainstay of management in both adult and pediatric RS. The medications frequently prescribed for the treatment of RS include antibiotics, intranasal steroids, and nasal saline [4]. Antibiotics are commonly used in pediatric and adult RS. In cases of acute bacterial sinusitis (ABS), a short course of 10-14 days of antibiotics is recommended [19]. However, in chronic rhinosinusitis (CRS), there is mixed data regarding the duration of antibiotics. Although several controlled trials did not show any benefit when using antibiotics for a short duration, some studies suggest giving a short course of three weeks when treating an exacerbation of CRS [15,20]. Some randomized controlled trials doubt the role of antibiotics in pediatric CRS as there is no difference in the outcome whether antibiotics are given or not [21-23]. In our study, approximately 50% of our patients received antibiotics. Regarding the use of intranasal corticosteroids (ICS), more than half of our patients used ICS. A systematic review and meta-analysis showed the benefits of using ICS in improving the symptoms and quality of life of patients and reducing the size of polyps in patients with CRS with polyposis [24,25]. In the pediatric age group, ICS are considered safe and tolerable. This is supported by a randomized clinical trial that assessed the safety of ICS [26]. Nevertheless, there is no substantiating proof to validate the effectiveness of intranasal steroids in addressing chronic RS among pediatric patients. Hence, the suggestion to employ ICS for managing pediatric chronic RS primarily relies on research conducted on adults [21]. There are other medications the patients received, with nasal saline and loratadine being the most common. Multiple studies suggest that the use of nasal saline treatment for both adult and pediatric CRS is effective for the clearance of thick nasal secretion [14,20,21]. Following the medical management, approximately 79% of patients improved while the rest did not show any improvement. Almost half of the patients who did not improve needed surgical management, with FESS being the most common (52.1%). The European Position Paper on Rhinosinusitis and Nasal Polyps recommends endoscopic surgery for adults [2]. On the other hand, there are apprehensions about the safety of endoscopic surgery in children. Nevertheless, there are studies suggesting the effectiveness of endoscopic surgery for patients who showed no improvement with medical treatment. A meta-analysis found that the success rate of endoscopic sinus surgery ranged from 71% to 100% [27].

Our study provides additional evidence regarding the prevalence and clinical characteristics of RS in pediatric patients. It included all RS patients in one of the largest tertiary centers in Saudi Arabia. Our study also provides a base for future research regarding RS-associated management options as well as the different symptomatology that patients may present with. Also, future studies can investigate the prevalence of RS using a control group to ensure a more accurate estimation of the real magnitude of this problem. The limitations of our study are the retrospective and observational nature along with being limited to a single center despite the relatively large sample size. In addition, the study has not assessed any other secondary outcomes. Moreover, only seven out of the 345 patients included in this study have done mucous culture, questioning our findings in this regard. Also, many patients have not maintained regular follow-ups, so we were not able to monitor their probability of developing any long-term complications, therefore, they were excluded from the study.

## Conclusions

In summary, RS is a prevalent condition that significantly impacts both our society and the well-being of children, compromising their quality of life. In our hospital, a majority of pediatric patients reported symptoms such as nasal obstruction, nasal discharge, and facial pain/discomfort. Chronic RS without nasal polyps was found to be the most frequent subtype, predominantly affecting the maxillary sinus. The primary approach for management involved medical therapy, resulting in significant improvement for the majority of patients. However, for those who did not experience improvement, surgical intervention was employed, with FESS being the prevailing procedure.
